# Clinical Relevance of Autoantibodies in Patients with Autoimmune Bullous Dermatosis

**DOI:** 10.1155/2012/369546

**Published:** 2012-12-24

**Authors:** Lilla Mihályi, Mária Kiss, Attila Dobozy, Lajos Kemény, Sándor Husz

**Affiliations:** Department of Dermatology and Allergology, Albert Szent-Györgyi Medical Center, University of Szeged, Korányi Fasor 6, 6720 Szeged, Hungary

## Abstract

The authors present their experience related to the diagnosis, treatment, and followup of 431 patients with bullous pemphigoid, 14 patients with juvenile bullous pemphigoid, and 273 patients with pemphigus. The detection of autoantibodies plays an outstanding role in the diagnosis and differential diagnosis. Paraneoplastic pemphigoid is suggested to be a distinct entity from the group of bullous pemphigoid in view of the linear C3 deposits along the basement membrane of the perilesional skin and the “ladder” configuration of autoantibodies demonstrated by western blot analysis. It is proposed that IgA pemphigoid should be differentiated from the linear IgA dermatoses. Immunosuppressive therapy is recommended in which the maintenance dose of corticosteroid is administered every second day, thereby reducing the side effects of the corticosteroids. Following the detection of IgA antibodies (IgA pemphigoid, linear IgA bullous dermatosis, and IgA pemphigus), diamino diphenyl sulfone (dapsone) therapy is preferred alone or in combination. The clinical relevance of autoantibodies in patients with autoimmune bullous dermatosis is stressed.

## 1. Introduction

The most frequent autoimmune bullous skin disorders are bullous pemphigoid (BP) and pemphigus vulgaris (PV). The diagnosis of both diseases relies not only on the clinical features but also on the detection of skin- or membrane-bound and circulating autoantibodies. We first diagnosed subepidermal bullous dermatosis in 1970 [1] by means of a direct immunofluorescence technique (DIF). We have subsequently examined, diagnosed, treated, and followed up several hundred patients with bullous skin diseases, and in this paper we present our experience in comparison with the literature findings.

## 2. Patients and Methods

Since 1970, we have diagnosed and treated 431 patients with BP (age range 38–102 years, mean 71.6 years), 14 children with juvenile BP (age range 3–14 years, mean 7.5 years), and 273 patients with pemphigus (age range 21–83 years, mean 53.9 years). All clinical investigations were conducted with the understanding and the consent of the patients. We are currently treating 47 patients with pemphigus and 45 with BP. The diagnoses were based on the clinical features and routine histological and immunohistological examinations [2]. For DIF tests, we used the intact skin adjacent to the bulla as substrate and antihuman IgG, IgA, IgM, and C3 conjugates labeled with FITC for antibody detection. For indirect immunofluorescence (IIF) examinations, we used esophagus samples from monkey and rabbit, and normal human skin; and for the salt split skin (SSS) tests, we applied normal human skin digested in 1.0 M NaCl solution [3]. Antibody detection was carried out with the same antihuman immunoglobulin (Ig) conjugates as for the DIF tests. The dilution of the sera was routinely 1 : 32. Western blot studies were performed according to Hashimoto et al., with slight modifications [4, 5]. The normal human skin pieces were incubated in 1.0 M NaCl at 4°C for 72 hours. The epidermis was then easily separated from the dermis and epidermis pieces were homogenized in a solution containing 31.2 mM Tris-HCl, 2% SDS, 1 mM PMSF, 2 mM EDTA, and 0.1 M dithiothreitol, and incubated for 24 hours at 4°C. The homogenizate was next centrifuged at 15000 g and the supernatants were stored at −70°C until use. The constituent proteins of the epidermal or dermal extracts were separated by SDS-PAGE (with 6% separating gel) and then transferred to nitrocellulose before probing with the test sera. All sera were used to probe immunoblots at a dilution of 1 : 40. Specific binding by the sera was detected by using peroxidase-linked class-specific second antibodies (goat antihuman IgG and IgA) and visualized with diaminobenzidine. For ELISA studies, antigenic epitopes of BP antigens were predicted by Peptide Structure and Plot Structure software, and the predicted peptides were chemically synthetized and screened with the use of serum from BP patients. The best antigenic epitopes were inserted as monomer and homo- and hetero-oligomer forms into fusion-expression plasmids inframe to the C-terminus of glutathione-S-transferase. Fusion products were expressed in *E. coli* cells and purified by affinity chromatography. The recombinant proteins were used [6, 7] for the detection of antibodies in the sera of BP subjects and controls (healthy persons or patients with PV or other bullous dermatoses). More recently, we have applied commercially available ELISA tests for the detection of the main autoantibody entities (MESACUP BP180 and BP230 tests desmoglein 1 (Dsg1) and desmoglein 3 (Dsg3) tests; MBL Medical and Biological Laboratories, Nagoya, Japan).

## 3. Results and Discussion

### 3.1. Autoantibodies in Pemphigoid

The diseases of the pemphigoid group are associated with tissue-bound and circulating autoantibodies against the protein components of hemidesmosomes. BP230 is an intracellular protein of 230-kDa that belongs to the plakin family of cytolinkers [8, 9]. The protein takes part in the linkage of the keratin filament network to the basal cell side [8–10]. The other autoantigen, BP180, is a transmembrane glycoprotein with extra- and intracellular parts. The extracellular portion consists of 15 collagenous and 16 noncollagenous subdomains and is also called collagen XVII [11]. The 16th noncollagenous domain (NC16A), which is localized just outside the cell membrane of the basal keratinocyte, is the immunodominant component of BP180. It serves as a cell-surface receptor, contributing to the maintenance of dermoepidermal cohesion by binding to laminin 5 [8]. BP180 is probably also involved in epidermal differentiation by facilitating the detachment of keratinocytes from the basal cell layer [12]. Autoantibodies against BP180 and BP230 mainly belong to subclasses IgG1, IgG4, and IgE. Antibodies against BP180 are responsible for the initial blisters, and anti-BP230 antibodies enhance the inflammatory reaction.

Autoantibodies in sera from epidermolysis bullosa acquisita (EBA) patients will bind to a 290-kDa band, the alfa chain of collagen VII, whereas this is not the case with sera from all other primary blistering diseases. A second band, of 145 kDa, will often be labeled with EBA antibodies. In most patients with linear IgA dermatoses (LABD), the recognized target antigens are constituted by 97-kDa or 120-kDa proteins, two fractions of BP180. LABD is characterized by circulating IgA antibodies against a 230-kDa antigen of dermal extracts, while in patients with IgA pemphigoid there is no reactivity against this antigen [13].

In mucous membrane pemphigoid (MMP), the target antigens vary; subsets of patients affected exclusively by oral and ocular mucosal diseases have autoantibodies targeting BP180, laminin332, *α*6*β*4 integrin, laminin311, and BP230 [14, 15]. Our investigations have demonstrated that *α*6 integrin antibodies can be identified in patients with BP who do not display mucous membrane symptoms in the oral cavity [16]. In herpes gestationis (HG), the target antigen is BP180 NC16A and antibody against BP230 can also be demonstrated.

### 3.2. Autoantibodies in Pemphigus

Most types of pemphigus antibodies are directed against the desmosomal cadherins known as Dsg1 and Dsg3. The PV antigen is a protein complex of 230 kDa which contains Dsg3 with a molecular weight of 130 kDa and plakoglobin with a molecular weight of 80 kDa. These two proteins are colligated by a disulfide bridge. Dsg3 is expressed primarily in the basal epidermal layers throughout the mucosa, while Dsg1 is expressed primarily in the upper levels of both the mucosa and the epidermis. Dsg3 alone is therefore able to maintain the mucosal integrity, and the impairment of Dsg1 does not cause mucosal lesions. If the patient has only mucocutaneous symptoms, anti-Dsg3 antibodies can be detected. In the event of skin and mucous membrane involvement, antibodies can be found against both Dsg1 and Dsg3. In PV there are always antibodies against Dsg3, while in pemphigus foliaceus (PF) only Dsg1 can be detected. In cases of paraneoplastic pemphigus, there are autoantibodies against some other desmosomal proteins, such as desmoplakin1, desmoplakin2, envoplakin, periplakin, plectin, BP230, BP180, and a not-further-characterized 170 kDa protein [17].

### 3.3. Diagnostic Possibilities in Patients with Autoimmune Blistering Diseases

The diagnosis of autoimmune blistering diseases can be confirmed by histological and immunopathological studies [18]. The exact diagnosis and the differential diagnosis rely on the cooperative work of the clinician and the histopathologist, but up-to-date laboratory tests are also indispensable. The localization of the bulla formation plays an important role in the diagnosis of these diseases.

**Figure 1:**
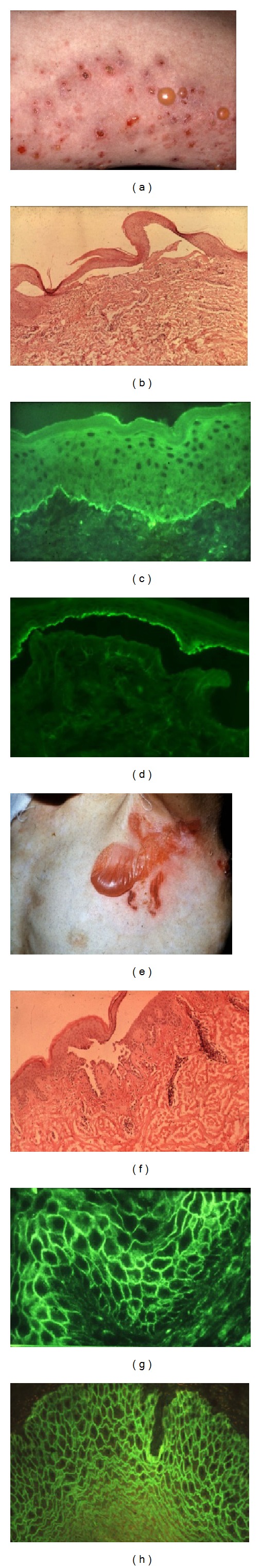
Bullous pemphigoid and pemphigus vulgaris. (a) The clinical picture of generalized pemphigoid with tense blisters, crusts, and erythematous plaques. (b) The histological picture of BP; subepidermal blister. (c) Linear basement zone deposits of IgG in BP. (d) Circulating IgG antibodies bind to the epidermal site of SSS in a patient with BP. (e) The flaccid blister in PV. (f) The histological picture of PV with intraepidermal blister. (g) DIF analysis demonstrates binding of C3 to the intercellular cement substance (ICS) in the upper stratum Malpighii. (h) An IIF study with monkey esophagus as substrate demonstrates the presence of anti-ICS antibody with antihuman IgG conjugate.

BP is most commonly seen in the elderly [19]. The lesions are tense blisters that occur either on healthy skin or on an erythematous base. The histological features of BP include a subepidermal blister with an inflammatory infiltrate that is often rich in eosinophils, but also contains lymphocytes, histiocytes, or neutrophils (Figure 1(b)). These can also be observed in several other related conditions, and therefore further diagnostic testing is essential. DIF investigations should be performed on healthy or erythematous nonbullous perilesional skin where linear basement membrane zone deposits of Ig-s (mainly IgG, and more rarely IgA, IgM, and IgE) and C3 are visible (Figure 1(c)). Similar DIF findings can also be seen in several other autoimmune blistering diseases, including EBA, cicatricial pemphigoid (CP), HG, and bullous eruption of systemic lupus erythematosus (VBSLE). IIF studies using SSS are therefore necessary for an appropriately complete evaluation of these patients [19]. SSS studies reveal that patients with BP and HG have circulating IgG antibodies attached to the epidermal side of the SSS (lamina lucida), while patients with EBA and VBSLE have circulating IgG antibodies that bind to the dermal side of the SSS (lamina densa and the anchoring fibril zone) (Figures 1(d) and 4(d)). Some patients with CP exhibit circulating IgG antibodies that bind to both the epidermal and the dermal side of the SSS preparation; these patients have the antilaminin-5 variant. An entity of the pemphigoid group is the lamina lucida type of linear IgA disease (IgA pemphigoid), characterized by IgA autoantibodies against BP180. Csorba et al. [20] strongly suggest that IgA pemphigoid and IgG BP are the two endpoints of the clinical spectrum of an immunological loss of tolerance against the components of hemidesmosomes, mediated by both IgG and IgA autoantibodies [20]. Previous studies have demonstrated that BP autoantibodies react predominantly with two distinct proteins of the hemidesmosomes, BP230, and BP180 [6]. Through western blotting, the presence of these two autoantibodies can be demonstrated. Western blot analysis can be useful in establishing the diagnosis of EBA and LABD. cDNAs of both BP autoantigens have been isolated and the amino acid sequences for these antigens have been deduced. From this point, several attempts have been made to diagnose bullous dermatosis with ELISA technology by the use of recombinant proteins [6, 7, 17, 21]. The sensitivity of our technique with ELISA assays of the serum of BP patients was 90% [6]. Other investigators who utilized different antigens and commercial kits reported very similar results [21]. However, the sensitivity can be increased up to 100% when various ELISA assays are applied to the NC16A domain and other extracellular portions of BP180 or BP230 or both. ELISA tests have nowadays largely replaced immunoblotting and immunoprecipitation techniques which are technically much more demanding. 

PV, the most common form of pemphigus, affects a younger population. PV can involve the skin and/or mucous membranes. Histological studies on cutaneous and mucosal biopsies reveal acantholysis in the suprabasilar part of the epidermis (Figure 1(f)). In PF, the blister formation is subcorneal. DIF studies typically demonstrate the binding of IgG and/or C3 to the intercellular cement substance (ICS) in the upper stratum Malpighii (Figure 1(g)). Both clinically and immunopathologically, IgA pemphigus is a unique entity. DIF studies have identified ICS reactions with IgA and C3. IIF studies with the use of epidermal substrate (monkey or rabbit esophagus) as substrate allow the detection of the presence of anti-ICS antibody with IgG and C3 in PV and PF, but with IgA and C3 in IgA pemphigus (Figure 1(h)). 

**Figure 2:**
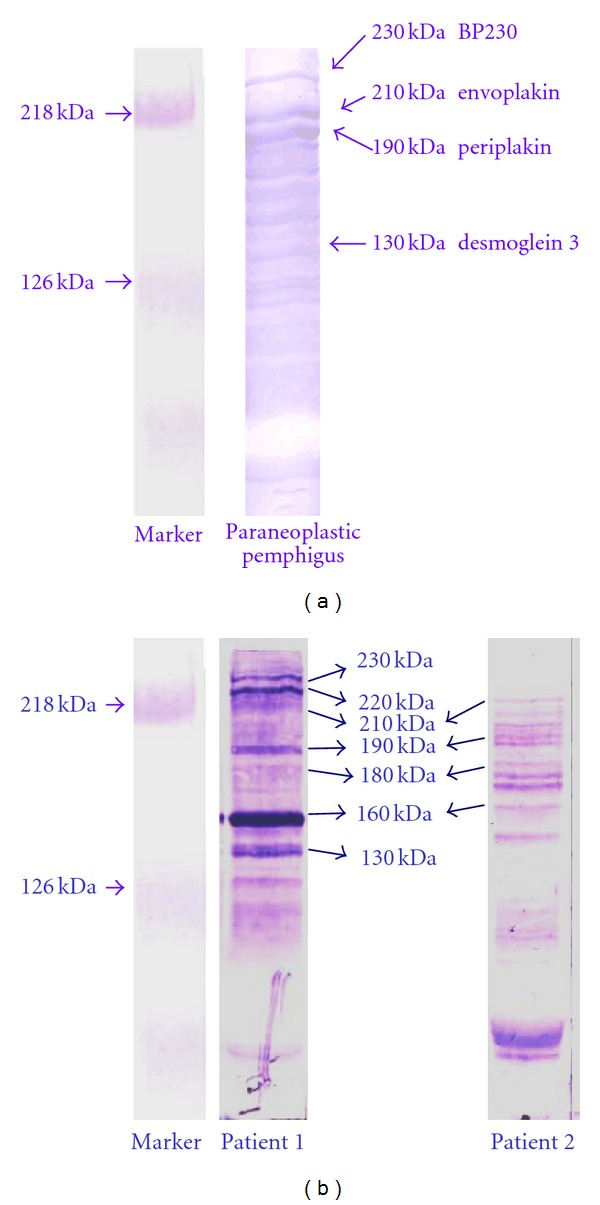
Western blot investigations. (a) A “ladder” configuration can be seen in paraneoplastic pemphigus. (b) A “ladder” configuration of paraneoplastic pemphigoid on western blot analysis.

The sera of patients with PV typically bind to a 130-kDa protein (Dsg3). The binding of sera to a 160-kDa protein (Dsg1) is seen in patients with PV, but to desmocollin 1 and to Dsg3 in patients with IgA pemphigus. Autoantibodies in paraneoplastic pemphigus typically target Dsg3 and proteins of the plakin family, including envoplakin, periplakin, plectin, desmoplakin, BP180, BP230, and a not-further-characterized 170-kDa protein [17]. In Western blot analysis, therefore we can see a “ladder” configuration (Figure 2(a)).

The recombinant ectodomains of Dsg1 and Dsg3 have been utilized to develop highly sensitive and specific ELISA assays. While the Dsg ectodomains are expressed in insect cells in two of the ELISA systems (MBI, Nagoya, Japan), the ectodomains in the other two available ELISA tests are generated in human HEK293 cells (Euroimmun, Lübeck, Germany) with a high potential of correct expression of conformational epitopes and the advantage that only the mature protein forms are expressed [17]. The ELISA systems are not only suitable for the diagnosis of the pemphigus group, but also useful in monitoring serum autoantibody levels during the course of the disease. In paraneoplastic pemphigus, an ELISA test based on the recombinant N-terminus of envoplakin and periplakin has been developed. It has been shown that the ELISA reactivity correlates with the disease activity of pemphigus patients.

### 3.4. Clinical Aspects of BP

BP diseases are subepidermal blistering illnesses with an incidence of 10 new cases per 1 million persons per year [19]. BP is characterized clinically by generalized, pruritic tense blisters, and crusts, usually on erythematous or apparently normal skin, together with infiltrated and urticarial plaques, papules, or eczematous lesions. The symptoms are most often symmetric and are located predominantly on the trunk and proximal extremities. Involvement of the oral cavity has been described in 10–30% of the cases [21]. BP sometimes begins with a nonbullous phase and nonspecific symptoms such as intense pruritus, excoriations, and urticarial or papular lesions, when several other diseases may be suspected, for example, eczema, a drug reaction, or chronic prurigo.

BP can be classified into two main groups: typical and atypical pemphigoid. In the typical type, generalized, localized, seborrheic, mucous membrane and paraneoplastic variants can be distinguished. Generalized pemphigoid is the most common form of the disease, with dozens to hundreds of blisters, usually affecting the elderly; the average age is 65 years (Figure 1(a)).

In most cases in the BP group we found the linear basement membrane zone deposition of IgG and C3 (365 patients), but in 20 patients linear basement membrane zone reactions with IgA and also with C3 were visible, and in 33 patients only C3, the third component of the complement could be observed. In 13 patients there was linear basement fluorescence with all Ig's and also with C3.

The localized form is characterized by some solitary eruptions on the head or on the extensor surface of the extremities, without causing complaints 

In certain cases of localized pemphigoid, it is not necessary to use systemic corticosteroids; topical corticosteroid therapy can lead to total recovery. 

 Seborrheic pemphigoid generally presents in older women with erythema and crusting in the seborrheic area [22].

In MMP, also known as CP, the oral cavity, conjunctiva, and laryngeal, nasal, or genital mucosa may be involved. With the exception of the oral mucosa, it usually heals with scars. In the mouth, desquamative gingivitis is the most frequent form. In cases of eye involvement, blindness can be the most serious complication, due to scarring.

Since the 1970s, we have had 37 patients with tumors (gastrointestinal, gynecological, urological, pulmonary, or endocrine) in BP. In some of these cases, the “ladder” configuration was seen on immunoblot analysis, between the typical 180 and 230 kDA bands of BP; the linear basement zone deposition of C3 was detected only with DIF (Figure 2(b)). The presence of a paraneoplastic type has been questioned in the literature in recent years. Some reports have indicated an increased frequency of certain cancers (such as digestive tract, lung) and lymphoproliferative disorders [23–26]. In contrast, other authors have concluded that there is no such connection [1, 27, 28].

In some of our patients, we observed linear basement zone deposits of IgA together with deposits of C3; in these cases, antibodies against BP180 and BP230 could be detected. We consider that this type should be distinguished from LABD, which is a unique entity both clinically and by western blot analysis.

The atypical group of pemphigoid includes LABD, linear IgM dermatosis, HG, VBSLE, EBA, and juvenile BP or drug-induced pemphigoid [29]. 

LABD is predilected on the face, especially periorally, on the scalp, and around the ears. Initially, pruritic, erythematous plaques and papules occur, which later display an annular or herpetiform pattern. New blisters may appear around an older lesion, forming a rosette-like shape [13]. We have encountered 2 patients with LABD. 

HG, which occurs most commonly in the third trimester of pregnancy and rarely in the postpartum period, causes intense pruritus. Many patients exhibit urticarial plaques, papules, or multiform lesions on the abdomen, but small herpetiform vesicles are rare, and differential diagnostic problems can therefore arise.

**Figure 3:**
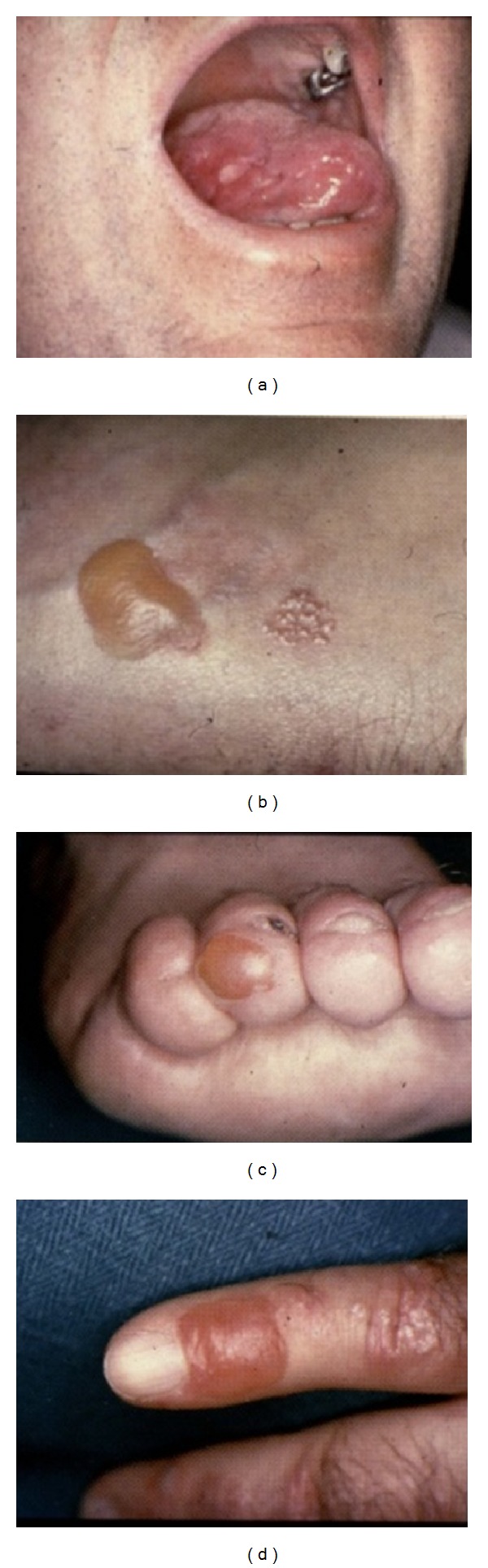
Epidermolysis bullosa aquisita. (a) Erosions in the oral cavity in EBA. (b) Blisters on the wrist in EBA. ((c) and (d)) Blisters on the fingertips in EBA.

In EBA, inflammation predominates at the beginning, after which scarring and fragility of the skin can be seen. The clinical signs are located at the pressure-exposed areas (Figures 3(a), 3(b), 3(c), and 3(d)). We have found 3 patients with EBA during the past 40 years. This is caused by IgG antibodies against type VII collagen [30, 31].

**Figure 4:**
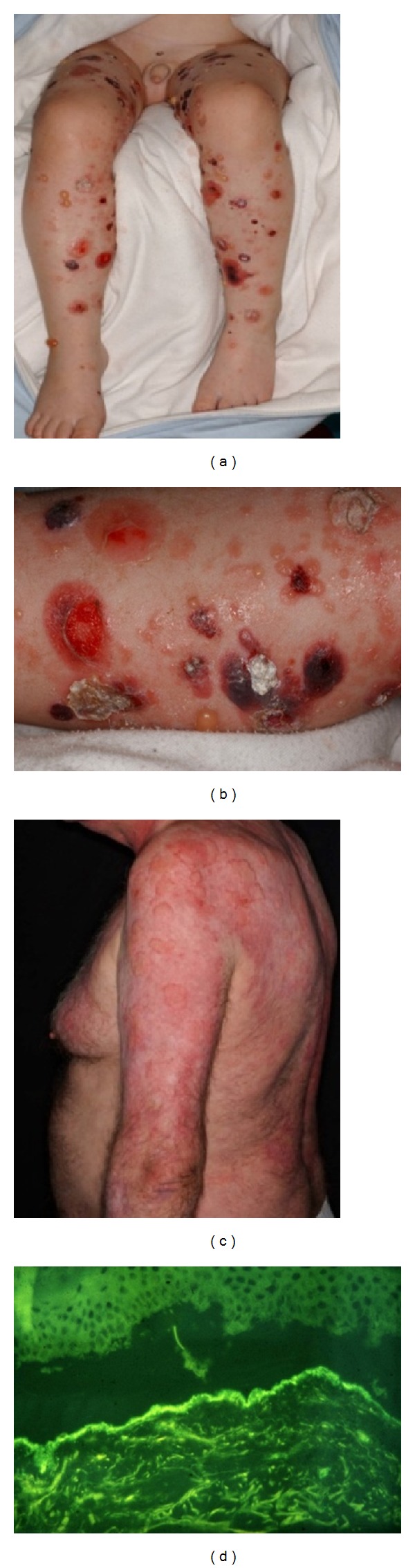
Juvenile bullous pemphigoid and bullous eruption of systemic lupus erythematosus. ((a) and (b)) Tense blisters and hemorrhagic crusts on the extremities of a 5-year- old boy with juvenile BP. (c) Erythematous plaques and some blisters in a VBSLE patient. (d) Circulating IgG antibodies bind to the dermal site of SSS in VBSLE.

Infants and children can also be affected; the signs of juvenile BP are similar to those of bullous impetigo (Figures 4(a) and 4(b)). We have treated 14 children with juvenile BP, most of whom exhibited linear basement deposits of IgA. The cause is unknown, though vaccination and drug intake may be assumed in some cases [32, 33].

Subjects with VBSLE have small grouped blisters on the light-exposed skin (Figure 4(c)), with antibodies against type VII collagen or laminin 5. Three of our patients also have SLE [34, 35] and in 1 patient Sjögren's syndrome is associated with bullous symptoms [36].

### 3.5. Clinical Aspects of Pemphigus Diseases

Diseases in the pemphigus group are rare, but often life threatening; they manifest intraepidermal blistering with suprabasal acantholysis. The various forms of pemphigus are differentiated on the basis of their clinical, immunopathological, and molecular biological features [2, 18]. They can be divided into two groups: typical and atypical pemphigus. Within the typical group, PV, PF, pemphigus seborrheicus (PS), pemphigus vegetans, and pemphigus erythematous (PE) can be distinguished.

The most common form is PV, which is characterized by extensive flaccid blisters, mucocutaneous erosions, and a hemorrhagic crust (Figure 1(e)). The mucocutaneous signs usually appear earlier than the skin problems; the content of the bulla is straw colored. The site of predilection is the face; while the areas of mechanical irritation such as the intertriginous regions, shoulders, elbows, buttocks, and back are also affected. The blisters tend to spread peripherally. PV occurs most often in the middle aged, generally starting with easily rupturing blister formation in an uninflamed skin area. The provoked Nikolsky' sign is positive. The skin lesions are less pruritic and often painful.

Pemphigus vegetans is suspected of being the infectious form of PV [37], where purulent plaques and granulomatous vegetation appear. Typical locations of pemphigus vegetans are the axilla, the perianal region, the genital tract, the nasolabial folds, or the scalp. Most of our cases proved to be PV, but we have had 9 patients with PF, 8 patients with PS, 2 patients with pemphigus herpetiformis (PH), 2 patients with paraneoplastic pemphigus, and 2 patients with pemphigus vegetans. 

PF is the superficial and less severe variant of PV [38]. The symptoms can be seen primarily on the scalp, the face, and the chest, as flaccid blisters which easily rupture and evolve into fine sheets of scales.

 PE, also called Senear-Usher disease, is similar to PF, but characterized by an additional “lupus band” of granular deposits of IgG and complement along the epidermal basement membrane zone. The clinical symptoms begin with erythematous patches on sun-exposed areas.

The atypical pemphigus group comprises drug-induced, paraneoplastic, IgA pemphigus and PH.

Drug-induced pemphigus can be caused by penicillamine, angiotensin-converting enzyme inhibitors, and pyrazolone derivatives. It displays the symptoms of PF or PE. 

In paraneoplastic pemphigus, there are polymorphic cutaneous lesions ranging from blisters to erosions, and even denudation on the trunk and the extremities, but also on the palms and soles. Severe, hemorrhagic, painful oral erosions are typical. This form tends to be associated with hematologic neoplasms. We have diagnosed 2 patients with this disease.

IgA pemphigus has two variants. The subcorneal pustular type is characterized by flaccid pustules which coalesce, leading to annular or polycyclic scaled lesions. The disease which is often associated with IgA gammopathy has a good prognosis. The second intraepidermal neutrophilic type is localized to the intertriginous regions and trunk. 

PH combines the clinical features of dermatitis herpetiformis with the immunopathological features of PV. The patients have small grouped vesicle and experience intense pruritus.

### 3.6. Therapy

For the treatment of autoimmune bullous diseases, we use immunosuppressive therapy and systemic corticosteroid as recommended in the literature [39]. In pemphigus we prescribe a higher dose (1.2 mg/body weight/day), and in pemphigoid a lower dose (0.75 mg/body weight/day) of corticosteroid, if necessary supplemented with azathioprine. Gastroprotective medication and potassium and calcium replacement are also regular. We gradually reduce the dosage of the immunosuppressants. In both pemphigus and bullous pemphigoid, when progression stops (usually after 2 weeks, when new blisters no longer appear), we reduce the corticosteroid dose, with alternation of administration of the starting dose and half of the starting dose every second day. Then, in line with the patient's clinical symptoms, we continue the reduction of the corticosteroid dose, usually at weekly intervals, maintaining the situation that the higher dose is always alternated with a dose that is 50% lower. When the higher dose reaches 30 mg/day, we reduce the lower dose to zero. The rate of dose reduction and the final dose can depend on the patient's status: faster in pemphigoid, and slower in pemphigus, during a period of some months or even years, until the definitive maintenance dose is reached. Finally, the maintenance corticosteroid dose (in pemphigoid generally 15–20 mg, and in pemphigus 25–30 mg corticosteroid) is administered only every second day. Thus the difference in the mode of reduction of the doses from the literature recommendations is that we reduce the corticosteroid dose intermittently by alternating between a lower and a higher corticosteroid dose, together with progressive dose reductions. Because of this, we observe fewer patients with severe corticosteroid side effects, the clinical signs can be kept in equilibrium, and even a symptom-free status can be achieved. 

It may occur that the corticosteroid therapy alone is not sufficient for the attainment of the complete remission. In these cases, and especially in pemphigus, we supplement the therapy with 2.5 mg/body weight/day of azathioprine. In severe or certain special cases, other medicaments and therapies may be suggested, in accordance with the literature (cytostatic drugs, intravenous Ig, and plasmapheresis). Novel targeted treatments such as immunoadsorption or rituximab, an anti-CD20 monoclonal antibody, have recently proven to be highly effective in severe and refractory autoimmune blistering diseases [40]. In patients with IgA autoantibodies we use diamino diphenyl sulfone therapy, alone or in combination.

## 4. Conclusions

 Our experience of more than 40 years with several hundred patients with autoimmune bullous disease may be summarized as follows.

1. In the BP group, DIF most frequently reveals linear deposits of IgG and C3 at the dermoepidermal junction, but sometimes linear basal membrane zone deposits of IgA and C3. These cases correspond to classical BP both clinically and by western blot assay, and in some of them circulating antibodies are detected with antihuman IgA conjugate on IIF analysis. We regard these cases as IgA pemphigoid. LABD, which also manifests the linear deposition of IgA, is a separate, unique entity with very low prevalence. Via the typical clinical symptoms and western blotting it can be well differentiated from IgA pemphigoid. We consider it is reasonable to list IgA pemphigoid in another group. Almost all of our juvenile BP cases were IgA pemphigoid. Diamino diphenyl sulfone has a role in the treatment of both diseases.
2. Regarding the literature argument as to whether BP is a paraneoplastic disease [1, 23–26, 28], we agree with those [27] who reject its paraneoplastic nature, because BP is a disease of the elderly, in whom malignant tumors appear more frequently irrespectively of BP. However, it is a fact and our cases also prove that systemic or local malignant tumors do occur in numerous cases. Immunological examination of these cases revealed that in most cases only linear C3 deposits were visible on DIF, with a “ladder-like” configuration on western blotting in some cases similarly as in paraneoplastic pemphigus. Following such an immunological result, a tumor search always yielded a positive result. Removal of the tumor influenced the prognosis of BP significantly, and the maintenance dose could be attained earlier. For these cases, we use the expression paraneoplastic pemphigoid.
3. The ELISA technique has practical importance in the primary diagnosis of both BP and pemphigus. Our investigations have indicated that the methodically chosen antigenic epitope or a combination of epitopes can substitute the total antigen structure immunologically and their production can be solved with the recombinant fusion technique [6, 7]. The usefulness of these antigenic epitopes is proved by the systematic provocation of BP-like symptom in mice [41]. Useful commercial antigens are currently available for the ELISA test.
4. With a new method for the treatment of both BP and pemphigus, the corticosteroid side effects were greatly reduced, without loss of the therapeutic effect. We suggest administration of the maintenance corticosteroid dose only every second day in both bullous diseases.
5. The autoantibodies have an outstanding role in both the diagnosis and the differential diagnosis. The detection of tissue-bound autoantibodies by DIF remains of great value in the diagnosis of bullous dermatoses; the ELISA technique is now playing a major part. The detection of circulating autoantibodies (IIF, western blotting, and SSS) is important from the aspect of the differential diagnosis of certain special disease forms.
6. In brief, the technique developed by Lever [42], and Jordon et al. [43, 44] in the mid-20th century for the diagnosis of autoimmune bullous diseases basically remains in place. With the subsequent refinements of that technique, all of the special forms of the various autoimmune bullous diseases can be unambiguously diagnosed.
